# Ethyl 6-amino-5-cyano-4-isopropyl-2-methyl-4*H*-pyran-3-carboxyl­ate

**DOI:** 10.1107/S160053680804052X

**Published:** 2008-12-06

**Authors:** Mehdi Messaâd, Besma Hamdi, Fakher Chabchoub, Abdelhamid Ben Salah, Mansour Salem

**Affiliations:** a Laboratoire de Chimie Appliquée: Hétérocycles, Corps Gras et Polymères, Faculté des Sciences de Sfax, BP 1171, 3000 Sfax, Tunisia; b Laboratoire des Sciences de Materiaux et d’Environnement, Faculté des Sciences de Sfax, BP 1171, 3000 Sfax, Tunisia

## Abstract

In the title compound, C_13_H_18_N_2_O_3_, the two H atoms of the NH_2_ group are engaged in hydrogen bonding with the N atom of the cyano group and with one O atom of the ethoxy­carbonyl group, building a chain parallel to the [100] direction. The N—H⋯N hydrogen bonds assemble the mol­ecules around inversion centres, forming dimers with an *R*_2_^2^(12) graph-set motif.

## Related literature

For general background, see: Messaâd *et al.* (2005 ▶, 2006 ▶); Mohr *et al.* (1975 ▶); Ohira & Yatagai (1993 ▶); Tandon *et al.* (1991 ▶); Wang *et al.* (1996 ▶); Zamocka *et al.* (1992 ▶); Bloxham *et al.* (1994 ▶); Elagamey *et al.* (1993 ▶); Khafagy *et al.* (2002 ▶). For graph-set notation, see: Etter (1990 ▶); Bernstein *et al.* (1994 ▶). 
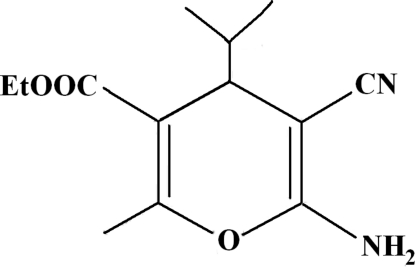


## Experimental

### 

#### Crystal data

C_13_H_18_N_2_O_3_
*M* *_r_* = 250.29
Triclinic, 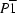

*a* = 8.0856 (1) Å
*b* = 9.3193 (2) Å
*c* = 10.4563 (2) Å
α = 65.652 (1)°
β = 69.679 (1)°
γ = 76.105 (1)°
*V* = 668.80 (2) Å^3^
*Z* = 2
Mo *K*α radiation
μ = 0.09 mm^−1^
*T* = 296 K
0.44 × 0.36 × 0.18 mm

#### Data collection

Bruker SMART CCD area-detector diffractometer
Absorption correction: multi-scan (*SADABS*; Bruker, 1998 ▶) *T*_min_ = 0.959, *T*_max_ = 0.982
17876 measured reflections
4664 independent reflections
3324 reflections with *I* > 2σ(*I*)
*R*_int_ = 0.027

#### Refinement

*R*[*F* ^2^ > 2σ(*F* ^2^)] = 0.046
*wR*(*F* ^2^) = 0.143
*S* = 1.05
4664 reflections
167 parameters
H-atom parameters constrained
Δρ_max_ = 0.24 e Å^−3^
Δρ_min_ = −0.24 e Å^−3^

### 

Data collection: *SMART* (Bruker, 1998 ▶); cell refinement: *SAINT* (Bruker, 1998 ▶); data reduction: *SAINT*; program(s) used to solve structure: *SHELXS97* (Sheldrick, 2008 ▶); program(s) used to refine structure: *SHELXL97* (Sheldrick, 2008 ▶); molecular graphics: *ORTEPIII* (Burnett & Johnson, 1996 ▶), *ORTEP-3 for Windows* (Farrugia, 1997 ▶) and *PLATON* (Spek, 2003 ▶); software used to prepare material for publication: *WinGX* (Farrugia, 1999 ▶).

## Supplementary Material

Crystal structure: contains datablocks I, global. DOI: 10.1107/S160053680804052X/dn2412sup1.cif

Structure factors: contains datablocks I. DOI: 10.1107/S160053680804052X/dn2412Isup2.hkl

Additional supplementary materials:  crystallographic information; 3D view; checkCIF report

## Figures and Tables

**Table 1:** Hydrogen-bond geometry (Å, °)

*D*—H⋯*A*	*D*—H	H⋯*A*	*D*⋯*A*	*D*—H⋯*A*
N2—H2*A*⋯O2^i^	0.86	2.08	2.9411 (11)	174
N2—H2*B*⋯N3^ii^	0.86	2.19	3.0269 (13)	164

Symmetry codes: (i) 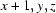; (ii) 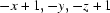.

**Fig. 1.**
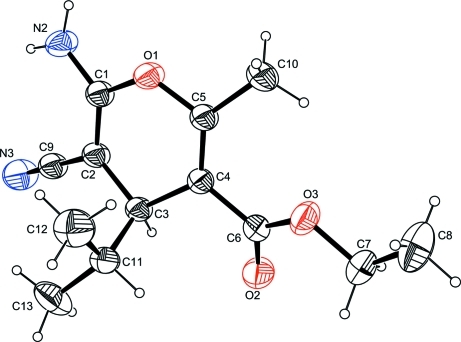
Molecular view of the title compound with the atom-labelling scheme. Ellispsoids are drawn at the 50% probability level. H atoms are represented as small spheres of arbitrary radii.

**Fig. 2.**
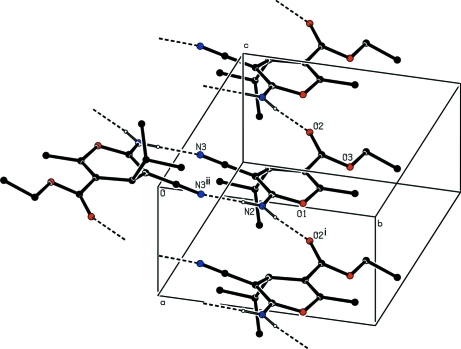
Partial packing view showing the formation of pseudo dimer through N—H···O and O—H···O hydrogen bonds. Hydrogen bonds are shown as dashed lines. [Symmetry codes: (i) 1 + x, y, z; (ii) 1 - x, -y, 1 - z]

**Fig. 3.**
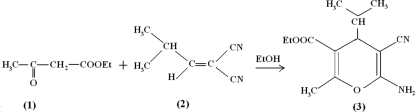
The formation of the title compound.

## supplementary crystallographic information

### Comment

The analysis of the bibliographical data shows that pyrans are biologically interesting compounds (Bloxham *et al.*, 1994; Wang *et al.*, 1996). In fact, some pyran derivatives present antibacterial activities (Zamocka *et al.*, 1992; Ohira & Yatagai, 1993); antifungal activities (Mohr *et al.*, 1975); antitumor activity (Tandon *et al.*, 1991) and they can have an hypotensive effect (Elagamey *et al.*, 1993). 2-amino-3-cyano-4*H*-pyrans are useful biphilic agents that lead to polycondensed pyranopyrimidines (Khafagy *et al.*, 2002; Messaâd *et al.*, 2005, 2006). In this paper we report for the first time the synthesis of 2-amino-3-cyano-5-ethoxycarbonyl-4-isopropyl-6-methyl-4*H*-pyran (3). This product was prepared *via* a standard addition of Michael of ethylacetoacetate (1) on α,β-ethylenic nitrile (2) in the presence of pyridine as a base (Scheme).

A view of the molecule is represented in Fig. 1. The two H atoms of the NH_2_ group are engaged in hydrogen bondings with the nitrogen of the cyano group and with one O atom of the ethoxy group then building a chain developing parallel to the [100] direction (Table 1, Fig. 2). The N—H···N hydrogen bonds assemble the molecules around inversion centres to form pseudo-dimers with a R_2_^2^(12) graph set motif (Etter, 1990; Bernstein *et al.*, 1994).

### Experimental

A mixture containing 1.3 g (0.01 mol) of ethylacetoacetate and 1.2 g (0.01 mol) of α,β-ethylenic nitrile in 50 ml of ethanol was heated to reflux for 3 h. The solvent was removed under rotary evaporation. The crude product was washed with ether then filtered and recrystallized from ethanol to give analytically pure crystals. Yield 75%; m.p. 118°C. Spectroscopic analysis, IR: νCN: 2183 cm-1; νNH2: 3334–3398 cm-1; νC═O:1692 cm-1; 1H NMR (300 MHz; CDCl3, p.p.m.): 1.29 (t, 3 J = 7.5, 3H); 4.21 (q, 3 J = 7.5, 2H); 2.29 (s, 1H); 4.48 (s, 2H); 3.37(d, 3 J = 4.5, 1H); 1.82 (m, 1H); 0.81–0.97 (2 d, 3 J = 9, 6H); 13 C NMR (75 MHz; CDCl3, p.p.m.): 14.16; 16.93; 18.27; 19.62; 34.58; 38.66; 57.20; 60.71; 108.42; 120.42; 157.64; 160.23; 166.62.

### Refinement

All H atoms attached to C atoms were fixed geometrically and treated as riding with C—H = 0.98 Å (C methine), 0.97 Å (C methylene), 0.96 Å (C methyl) and 0.86 Å (NH) with *U*_iso_(H) = 1.2 *U*_eq_(C methine, C methylene and NH) and *U*_iso_(H) = 1.5 *U*_eq_(C methyl).

In the absence of significant anomalous scattering, the absolute configuration could not be reliably determined and then the Friedel pairs were merged and any references to the Flack parameter were removed.

### Crystal data

**Table:**

C_13_H_18_N_2_O_3_	*Z* = 2
*M**_r_* = 250.29	*F*(000) = 268
Triclinic, *P*1	*D*_x_ = 1.243 Mg m^−^^3^
Hall symbol: -P 1	Mo *K*α radiation, λ = 0.71073 Å
*a* = 8.0856 (1) Å	Cell parameters from 2132 reflections
*b* = 9.3193 (2) Å	θ = 2.3–21.2°
*c* = 10.4563 (2) Å	µ = 0.09 mm^−^^1^
α = 65.652 (1)°	*T* = 296 K
β = 69.679 (1)°	Prism, colourless
γ = 76.105 (1)°	0.44 × 0.36 × 0.18 mm
*V* = 668.80 (2) Å^3^	

### Data collection

**Table:**

Bruker SMART CCD area-detector diffractometer	4664 independent reflections
Radiation source: sealed tube	3324 reflections with *I* > 2σ(*I*)
graphite	*R*_int_ = 0.027
φ and ω scans	θ_max_ = 32.1°, θ_min_ = 2.2°
Absorption correction: multi-scan (*SADABS*; Bruker, 1998)	*h* = −12→12
*T*_min_ = 0.959, *T*_max_ = 0.982	*k* = −13→12
17876 measured reflections	*l* = −15→14

### Refinement

**Table:**

Refinement on *F*^2^	Primary atom site location: structure-invariant direct methods
Least-squares matrix: full	Secondary atom site location: difference Fourier map
*R*[*F*^2^ > 2σ(*F*^2^)] = 0.046	Hydrogen site location: inferred from neighbouring sites
*wR*(*F*^2^) = 0.143	H-atom parameters constrained
*S* = 1.05	*w* = 1/[σ^2^(*F*_o_^2^) + (0.0801*P*)^2^ + 0.026*P*] where *P* = (*F*_o_^2^ + 2*F*_c_^2^)/3
4664 reflections	(Δ/σ)_max_ = 0.009
167 parameters	Δρ_max_ = 0.24 e Å^−^^3^
0 restraints	Δρ_min_ = −0.24 e Å^−^^3^

### Special details

**Table:**

Geometry. All esds (except the esd in the dihedral angle between two l.s. planes) are estimated using the full covariance matrix. The cell esds are taken into account individually in the estimation of esds in distances, angles and torsion angles; correlations between esds in cell parameters are only used when they are defined by crystal symmetry. An approximate (isotropic) treatment of cell esds is used for estimating esds involving l.s. planes.
Refinement. Refinement of *F*^2^ against ALL reflections. The weighted *R*-factor *wR* and goodness of fit *S* are based on *F*^2^, conventional *R*-factors *R* are based on *F*, with *F* set to zero for negative *F*^2^. The threshold expression of *F*^2^ > σ(*F*^2^) is used only for calculating *R*-factors(gt) *etc*. and is not relevant to the choice of reflections for refinement. *R*-factors based on *F*^2^ are statistically about twice as large as those based on *F*, and *R*- factors based on ALL data will be even larger.

### Fractional atomic coordinates and isotropic or equivalent isotropic displacement parameters (Å^2^)

**Table:**

	*x*	*y*	*z*	*U*_iso_*/*U*_eq_	
O1	0.36662 (8)	0.54982 (7)	0.29951 (8)	0.03977 (18)	
O2	−0.23592 (10)	0.56365 (10)	0.34370 (11)	0.0559 (2)	
O3	−0.12340 (9)	0.79434 (9)	0.22760 (9)	0.0463 (2)	
N2	0.54618 (11)	0.33268 (10)	0.37642 (11)	0.0440 (2)	
H2A	0.6161	0.3972	0.3631	0.053*	
H2B	0.5773	0.2321	0.4080	0.053*	
N3	0.29885 (15)	0.00436 (11)	0.48430 (13)	0.0583 (3)	
C1	0.38924 (12)	0.38872 (11)	0.34801 (10)	0.0340 (2)	
C2	0.26177 (12)	0.30627 (10)	0.36205 (10)	0.0341 (2)	
C3	0.10119 (11)	0.39019 (10)	0.30733 (10)	0.03172 (19)	
H3	−0.0020	0.3380	0.3801	0.038*	
C4	0.07103 (11)	0.55961 (10)	0.29910 (10)	0.03243 (19)	
C5	0.19954 (12)	0.63057 (10)	0.29517 (10)	0.0344 (2)	
C6	−0.11029 (12)	0.63756 (12)	0.29492 (11)	0.0364 (2)	
C7	−0.29880 (14)	0.87305 (14)	0.21657 (15)	0.0535 (3)	
H7A	−0.3819	0.8524	0.3133	0.064*	
H7B	−0.3409	0.8342	0.1618	0.064*	
C8	−0.2844 (2)	1.04593 (17)	0.14025 (19)	0.0738 (4)	
H8A	−0.2495	1.0843	0.1982	0.111*	
H8B	−0.3973	1.1007	0.1264	0.111*	
H8C	−0.1972	1.0644	0.0469	0.111*	
C9	0.28332 (13)	0.13998 (11)	0.42860 (12)	0.0393 (2)	
C10	0.19803 (15)	0.79274 (12)	0.28995 (14)	0.0471 (3)	
H10A	0.0785	0.8439	0.3048	0.071*	
H10B	0.2700	0.8531	0.1963	0.071*	
H10C	0.2446	0.7859	0.3654	0.071*	
C11	0.11157 (12)	0.38374 (12)	0.15880 (11)	0.0382 (2)	
H11	0.0071	0.4499	0.1297	0.046*	
C12	0.27420 (17)	0.45164 (17)	0.03829 (13)	0.0573 (3)	
H12A	0.3795	0.3903	0.0639	0.086*	
H12B	0.2728	0.5596	0.0266	0.086*	
H12C	0.2730	0.4479	−0.0518	0.086*	
C13	0.1026 (2)	0.21704 (15)	0.17254 (16)	0.0614 (3)	
H13A	0.0852	0.2204	0.0850	0.092*	
H13B	0.0054	0.1728	0.2548	0.092*	
H13C	0.2116	0.1525	0.1865	0.092*	

### Atomic displacement parameters (Å^2^)

**Table:**

	*U*^11^	*U*^22^	*U*^33^	*U*^12^	*U*^13^	*U*^23^
O1	0.0361 (3)	0.0241 (3)	0.0613 (4)	−0.0051 (2)	−0.0227 (3)	−0.0092 (3)
O2	0.0355 (4)	0.0475 (5)	0.0925 (6)	−0.0084 (3)	−0.0162 (4)	−0.0320 (4)
O3	0.0353 (3)	0.0343 (4)	0.0665 (5)	0.0028 (3)	−0.0207 (3)	−0.0140 (3)
N2	0.0393 (4)	0.0292 (4)	0.0672 (6)	−0.0028 (3)	−0.0279 (4)	−0.0109 (4)
N3	0.0649 (6)	0.0298 (5)	0.0844 (8)	−0.0039 (4)	−0.0399 (6)	−0.0098 (5)
C1	0.0358 (4)	0.0255 (4)	0.0421 (5)	−0.0041 (3)	−0.0164 (4)	−0.0088 (3)
C2	0.0378 (4)	0.0246 (4)	0.0437 (5)	−0.0043 (3)	−0.0185 (4)	−0.0100 (3)
C3	0.0322 (4)	0.0248 (4)	0.0417 (4)	−0.0057 (3)	−0.0146 (3)	−0.0106 (3)
C4	0.0320 (4)	0.0264 (4)	0.0424 (5)	−0.0030 (3)	−0.0143 (3)	−0.0128 (3)
C5	0.0357 (4)	0.0258 (4)	0.0450 (5)	−0.0038 (3)	−0.0171 (4)	−0.0111 (3)
C6	0.0334 (4)	0.0343 (5)	0.0481 (5)	−0.0018 (3)	−0.0138 (4)	−0.0204 (4)
C7	0.0398 (5)	0.0505 (7)	0.0749 (8)	0.0106 (5)	−0.0261 (5)	−0.0276 (6)
C8	0.0675 (8)	0.0544 (8)	0.0838 (10)	0.0189 (6)	−0.0321 (7)	−0.0150 (7)
C9	0.0413 (5)	0.0289 (5)	0.0525 (5)	−0.0034 (4)	−0.0224 (4)	−0.0120 (4)
C10	0.0509 (5)	0.0294 (5)	0.0710 (7)	−0.0048 (4)	−0.0277 (5)	−0.0188 (5)
C11	0.0393 (5)	0.0369 (5)	0.0472 (5)	−0.0021 (4)	−0.0200 (4)	−0.0183 (4)
C12	0.0578 (7)	0.0678 (8)	0.0461 (6)	−0.0139 (6)	−0.0110 (5)	−0.0195 (6)
C13	0.0818 (9)	0.0518 (7)	0.0718 (8)	−0.0148 (6)	−0.0272 (7)	−0.0343 (6)

### Geometric parameters (Å, °)

**Table:**

O1—C1	1.3599 (11)	C7—C8	1.4871 (18)
O1—C5	1.3855 (11)	C7—H7A	0.9700
O2—C6	1.2053 (11)	C7—H7B	0.9700
O3—C6	1.3308 (12)	C8—H8A	0.9600
O3—C7	1.4516 (12)	C8—H8B	0.9600
N2—C1	1.3367 (11)	C8—H8C	0.9600
N2—H2A	0.8600	C10—H10A	0.9600
N2—H2B	0.8600	C10—H10B	0.9600
N3—C9	1.1489 (13)	C10—H10C	0.9600
C1—C2	1.3625 (12)	C11—C13	1.5172 (15)
C2—C9	1.4077 (13)	C11—C12	1.5194 (15)
C2—C3	1.5113 (12)	C11—H11	0.9800
C3—C4	1.5091 (12)	C12—H12A	0.9600
C3—C11	1.5513 (13)	C12—H12B	0.9600
C3—H3	0.9800	C12—H12C	0.9600
C4—C5	1.3404 (11)	C13—H13A	0.9600
C4—C6	1.4785 (12)	C13—H13B	0.9600
C5—C10	1.4868 (13)	C13—H13C	0.9600
			
C1—O1—C5	119.76 (7)	C7—C8—H8A	109.5
C6—O3—C7	116.29 (8)	C7—C8—H8B	109.5
C1—N2—H2A	120.0	H8A—C8—H8B	109.5
C1—N2—H2B	120.0	C7—C8—H8C	109.5
H2A—N2—H2B	120.0	H8A—C8—H8C	109.5
N2—C1—O1	110.47 (7)	H8B—C8—H8C	109.5
N2—C1—C2	128.56 (8)	N3—C9—C2	179.11 (13)
O1—C1—C2	120.97 (8)	C5—C10—H10A	109.5
C1—C2—C9	118.33 (8)	C5—C10—H10B	109.5
C1—C2—C3	121.20 (8)	H10A—C10—H10B	109.5
C9—C2—C3	120.47 (7)	C5—C10—H10C	109.5
C4—C3—C2	109.19 (7)	H10A—C10—H10C	109.5
C4—C3—C11	110.66 (7)	H10B—C10—H10C	109.5
C2—C3—C11	114.29 (8)	C13—C11—C12	110.81 (10)
C4—C3—H3	107.5	C13—C11—C3	111.65 (9)
C2—C3—H3	107.5	C12—C11—C3	112.53 (8)
C11—C3—H3	107.5	C13—C11—H11	107.2
C5—C4—C6	124.13 (8)	C12—C11—H11	107.2
C5—C4—C3	121.99 (8)	C3—C11—H11	107.2
C6—C4—C3	113.88 (7)	C11—C12—H12A	109.5
C4—C5—O1	120.88 (8)	C11—C12—H12B	109.5
C4—C5—C10	130.87 (9)	H12A—C12—H12B	109.5
O1—C5—C10	108.23 (7)	C11—C12—H12C	109.5
O2—C6—O3	122.42 (8)	H12A—C12—H12C	109.5
O2—C6—C4	122.32 (9)	H12B—C12—H12C	109.5
O3—C6—C4	115.19 (7)	C11—C13—H13A	109.5
O3—C7—C8	107.55 (10)	C11—C13—H13B	109.5
O3—C7—H7A	110.2	H13A—C13—H13B	109.5
C8—C7—H7A	110.2	C11—C13—H13C	109.5
O3—C7—H7B	110.2	H13A—C13—H13C	109.5
C8—C7—H7B	110.2	H13B—C13—H13C	109.5
H7A—C7—H7B	108.5		
			
C5—O1—C1—N2	−165.46 (8)	C6—C4—C5—C10	1.72 (18)
C5—O1—C1—C2	14.55 (14)	C3—C4—C5—C10	−178.70 (10)
N2—C1—C2—C9	7.28 (17)	C1—O1—C5—C4	−18.29 (14)
O1—C1—C2—C9	−172.73 (9)	C1—O1—C5—C10	160.34 (9)
N2—C1—C2—C3	−172.47 (10)	C7—O3—C6—O2	0.41 (16)
O1—C1—C2—C3	7.52 (15)	C7—O3—C6—C4	177.50 (9)
C1—C2—C3—C4	−23.01 (13)	C5—C4—C6—O2	−155.83 (11)
C9—C2—C3—C4	157.24 (9)	C3—C4—C6—O2	24.56 (14)
C1—C2—C3—C11	101.52 (11)	C5—C4—C6—O3	27.08 (14)
C9—C2—C3—C11	−78.22 (11)	C3—C4—C6—O3	−152.53 (9)
C2—C3—C4—C5	19.51 (13)	C6—O3—C7—C8	179.26 (11)
C11—C3—C4—C5	−107.12 (10)	C4—C3—C11—C13	−167.94 (8)
C2—C3—C4—C6	−160.87 (8)	C2—C3—C11—C13	68.32 (10)
C11—C3—C4—C6	72.50 (9)	C4—C3—C11—C12	66.72 (11)
C3—C4—C5—O1	−0.42 (14)	C2—C3—C11—C12	−57.03 (11)

### Hydrogen-bond geometry (Å, °)

**Table:**

*D*—H···*A*	*D*—H	H···*A*	*D*···*A*	*D*—H···*A*
N2—H2A···O2^i^	0.86	2.08	2.9411 (11)	174
N2—H2B···N3^ii^	0.86	2.19	3.0269 (13)	164

Symmetry codes: (i) *x*+1, *y*, *z*; (ii) −*x*+1, −*y*, −*z*+1.
